# “Dr. Google” consultation and health anxiety among primary healthcare patients: The mediation effect of health literacy

**DOI:** 10.1371/journal.pone.0325791

**Published:** 2025-10-23

**Authors:** Eddieson Pasay-an, Reem Humaidi Alalawi, Sumathi Robert Shanmugam, Salman Amish Alshammari, Maha Sanat Alreshidi, Sameer Alkubati, Nojoud Alrashidi, Petelyne Pangket, Lizy Sonia Benjamin, Ferdinand Gonzales, Lailani Sacgaca, Romeo Mostoles Jr

**Affiliations:** 1 College of Nursing, King Khalid University, Abha, Saudi Arabia; 2 Department of Maternity and Pediatric Nursing, College of Nursing, Princess Nourah bint Abdulrahman University, Riyadh, Saudi Arabia; 3 King Khalid Hospital, Hail City, Saudi Arabia; 4 College of Nursing, University of Hail, Hail City, Saudi Arabia; 5 College of Nursing, Taif University, Taif, Saudi Arabia; De La Salle University, PHILIPPINES

## Abstract

**Background:**

Promoting health literacy is a successful intervention for cyberchondriasis and health anxiety. However, no study has examined how cyberchondria, health anxiety, and health literacy are interrelated.

**Objective:**

This study aimed to determine the prevalence of cyberchondria among primary healthcare patients in Saudi Arabia and examine the relationship between cyberchondria and health anxiety and the mediating role of health literacy in this relationship.

**Methods:**

This cross-sectional study involved 422 participants from all over Saudi Arabia’s five regions, specifically from primary healthcare centers. Data were collected between August 1, 2024 to September 1, 2024.

**Results:**

Age, sex, marital status, nationality, region, place of residence, social media use, and Internet use were significantly associated with cyberchondria health literacy and health anxiety (p < 0.001). Structural equation modeling (SEM) revealed that cyberchondria had a positive effect on both health literacy and health anxiety (p < 0.001). Additionally, regarding the age group 65 years or older, for example, results indicate that in accordance with most previous studies reviewed here, cyberchondria can predict higher levels of health anxiety in people who have low levels of education.

**Conclusions:**

Cyberchondria is interrelated with other variables such as demographic characteristics and behavioral patterns. They highlighted the significance of interventions directed at enhancing health literacy to curtail cyberchondria and reduce health anxiety. The present findings suggest that cyberchondria manifests in many different ways, making it a complex phenomenon. It also helps us to understand how the patient may become an active partner rather than a passive recipient in his/her own care.

## Introduction

The rapid increase in the number of users visiting health sites betrays the rise of the Internet as the most sought-after source for medical information [1]. It is a useful tool for self-sufficient individuals to achieve maximum well-being. However, it can be significantly distressing among vulnerable people [2,3]. Although having access to online data is advantageous in numerous ways, it also risks overloading the public with medical information [4]. Such excessive knowledge acquisition usually leads to heightened anxiety and unending need for further medical details, giving rise to a phenomenon referred to as “cyberchondria” [5], which is the most common subclinical condition involving persistent or repetitive internet searching for health-related materials along with anticipatory worry about one’s own health. Lee et al. [6] described this behavior as “Dr. Google,” where people use Google’s self-care searches to find answers about their illness. For this reason, we use the terms “cyberchondria” and “Dr. Google” interchangeably throughout this discussion.

Recent studies have found that people do not primarily use the Internet to search for health-related information to replace medical attention but to learn more about their own issues before meeting doctors [7,8]. This trend is especially noticeable among older generations, who tend to worry much more than younger ones after perusing information about sicknesses online [9]. Additionally, research has shown that individuals who have little or no background knowledge in medicine are liable to cyberchondria as they explore the web in search of advice for themselves or others [10,11]. Moreover, most of them were young women who were educated [12].

“Health anxiety” is a psychological condition characterized by the amplification of physical and mental concerns triggered by perceived threats to one’s health [13]. A growing body of research shows that individuals who seek health-related information online often experience heightened levels of nervousness and depression, while dedicating substantial amounts of time to this pursuit, thereby fostering health anxiety. The primary motivators for accessing the Internet to search for health-related information are anxiety and the need for reassurance [1]. Consequently, the severity of physiological symptoms and perception of danger serve as catalysts for engaging in online activities [1]. As a result, individuals plagued by persistent concerns regarding severe medical conditions find their daily lives detrimentally impacted by their search behavior [14], ultimately compromising their overall health-related quality of life [15]. In fact, it has been found that individuals with high levels of health anxiety frequently misinterpret their physical symptoms as indications of serious illnesses [16], with young adults reporting higher levels of health anxiety than older individuals [17].

Health literacy (HL) is gaining recognition as a strategy for enhancing individual health outcomes and self-management [18–20]. Achieving and maintaining optimal health outcomes within individual and system contexts requires individuals to access and comprehend their knowledge and information [21]. This includes acquiring the necessary skills and capabilities to seek, analyze, evaluate, and apply health-related information. Such competencies are essential for making informed judgments and decisions regarding routine healthcare, disease prevention, and health promotion activities to enhance quality of life over the course of a lifetime [22,23].

The World Health Organization [24] notes that HL enables people to take care of themselves, participate in community health programs with others, and hold the government responsible for resolving health issues in society. Indeed, it is not patients’ socio-demographic characteristics, but rather their level of HL, that most strongly predicts their final health outcomes [25]. Additionally, there is evidence that HL can help control cyberchondria and health anxiety. This strategy can mitigate the second-hand effects of people’s emotional reactions to cyberchondria and anxiety [26]. While not all people are prone to the negative consequences of “visiting Dr. Google” on health anxiety, those who are vulnerable may experience increased stress when using online sources of health information. Previous studies on HL among cyberchondriacs have yet to examine its relationship with health anxiety.

Concerns have been raised in Saudi Arabia about a growing overdependence on Internet platforms for health information, which has given rise to “cyberchondria,” the habit of excessive online searches leading to increased health fears. In one such instance, El-Zayat et al. (2023) [27] investigated the association between cyberchondria and mobile phone addiction, as well as electronic health literacy among Saudi Arabian youth, in order to establish that many of them suffer from cyberchondria. Likewise, Almansef (2021) demonstrated that mental illiteracy and medical illiteracy are associated with better outcomes in terms of mental well-being. Henceforth, this knowledge will be crucial for designing interventions that are specific to the three aspects needed to finally resolve the problem of excessive internet search for medical information by Saudis to improve health in general terms. When it comes to finding solutions to the rampant Internet search for healthcare information, a clear understanding of how cyberchondria, mental literacy, and health literacy interplay is essential. Previous studies have shown links between cyberchondria and health anxiety, whereas people who can read about their own conditions tend not to fear it much.

Understanding the interplay between these variables is crucial for developing interventions to address the challenges associated with excessive online health information-seeking and improving overall health outcomes in the Saudi population. This study aimed to determine the prevalence of cyberchondria among primary healthcare patients in Saudi Arabia; examine the relationship between cyberchondria, health anxiety, and health literacy in the Saudi context; investigate the mediating role of health literacy in the relationship between cyberchondria and health anxiety; and inform interventions aimed at reducing the negative impacts of cyberchondria on mental health and promoting healthier information consumption habits in Saudi Arabia.

This study holds particular relevance as it aims to consolidate existing knowledge on the emerging concepts of cyberchondria and health anxiety among primary healthcare clients, benefiting healthcare professionals. Treating cyberchondriasis and health anxiety requires a comprehensive approach based on a deeper logical and empirical understanding of these issues. The findings can inform policymakers on how best to address these concerns by synthesizing current knowledge on the phenomenon of relying on online resources like “Dr. Google.” Solving such complex problems requires a comprehensive strategy supported by thorough logical reasoning and empirical comprehension.

## Methods

### Research design

This study used a descriptive cross-sectional design. This design involved the collection of data within a single point in time to examine the prevalence and relationships between cyberchondria, health anxiety, and health literacy in a Saudi population sample.

### Participants/Setting

This study was conducted in five main regions of Saudi Arabia, at primary healthcare facilities that serve a population of 2,0000,000 individuals. The number of participants required for the study was established using a RAOSOFT calculator based on a metropolitan population of 32 million [28]. Primary healthcare centers were chosen as the setting for this study, considering their accessibility and representation of the general population. These centers provide an ideal platform to cover a range of individuals who usually seek healthcare services. The aim of recruiting participants from primary care settings was to obtain a sample that represented a larger population, thereby improving generalizability. A total of 385 respondents were recruited using convenience sampling. A sample size increase of 10% was performed to allow for any contingencies, such as nonresponse or possible dropouts due to non-random selection, thus giving us a final sample size of 424 persons. Eligible participants included clients visiting primary healthcare facilities for routine check-ups. Those aged 18 years and older with reading skills and understanding the English language as well as being ready to consent took part in the study.

### Data collection

Data collection commenced only after ethical approval was obtained. Participants were informed about the study’s objective, their level of participation, and their rights as participants. During their visits to primary healthcare facilities, a paper-format questionnaire was distributed among them. They were required to read and understand the informed consent form before answering the questions. Participants had not spent less than 15 minutes but could spend more time if they wished to freely complete the survey at their own pace. Data were collected between August 1,2024 to September 1,2024.

### Questionnaire

Three specific instruments were used to evaluate different aspects. First, the Cyberchondria Severity Scale’s Short Form (CSS-12) was used to assess the severity of the condition [26]. It consists of 12 items measured on a five-point Likert-type scale with four subscales. The CSS-12 subscale consists of items 1, 3, and 6, while the reassurance subscale consists of items 2, 7, 11, and 10. Finally, CSS-12 includes Items 4, 8, and 9. The total score ranged from 12 to 60. The CSS-12 has demonstrated reliable results, with Cronbach’s alphas for its subscales ranging between 0.73 and 0.90 [29].

Second, health anxiety was evaluated using the Short Health Anxiety Inventory (SHAI) without considering physical health [30]. There are 18 items in the inventory scored as a composite (0–54) and in two subscales: Health Anxiety (items 1–14, range 0–42), which assesses distress about health, and Negative Consequences of becoming ill (items 15–18, range 0–12). Increased scores signify increased anxiety over health or infirmity.

Finally, the BRIEF Health Literacy Screening Tool was used. It was developed by Haun et al. [31] and consists of a validated four-item screening questionnaire aimed at assessing patients’ understanding of medical information. The tool includes questions regarding the frequency of having someone read hospital records together or difficulty understanding written information about one’s medical condition. The participants responded to each question using a five-point scale. Scores ranged from 2 to 20, with different ranges indicating inadequate, marginal, and adequate HL. BRIEF is a more time-efficient and less embarrassing alternative to the original questionnaire.

These questionnaires were carefully validated and examined for reliability in a local context. Two researchers and two psychometricians with doctoral degrees in philosophy conducted the validation process. A reliability test was performed on 15 participants, resulting in good reliability scores for HL (0.83), CSS (0.79), and health anxiety (0.80).

### Ethical consideration

This study adhered to ethical principles and guidelines. Informed written consent was obtained from all participants prior to their involvement in the study. The consent form outlined the study’s objectives, procedures, potential risks and benefits, and participants’ right to withdraw at any time. The informed consent process was documented by obtaining signed consent forms from each participant.

The study protocol was reviewed and approved by the Institutional Review Board of the University of Hail (5336/5/43). Participants were assured that all collected information would be treated with utmost confidentiality.

### Statistical treatment

Descriptive variables were characterized using frequencies and percentages in SPSS version 26. Structural equation modeling (SEM) was conducted using AMOS 21.0 to assess the mediating role of HL in the relationship between cyberchondria and health anxiety. The model was estimated using the maximum likelihood estimation approach, and several indices were used to evaluate goodness of fit, including CMIN/DF, RMSEA, SRMR, and CFI. The moderating effect of age was evaluated using Hayes’ SPSS-PROCESS program model 59 in a multivariate regression analysis involving age, HL, cyberchondriasis, and health anxiety [32]. Finally, a bootstrapped approach with 5000 bootstraps and a 95% confidence interval was used to estimate the importance of the indirect effects. Statistical significance was set at P-values < 0.05.

## Results

Table 1 presents respondents’ demographic characteristics. Of the 424 participants, most were less than 30 years old (39.4%), female (53.4%), single (51.2%), and Saudi (72.2%). Most of the respondents resided in urban areas (84.2%). Riyadh had the largest share of participants (28.3%). When asked if they were frequent social media users, 78.5% answered yes and 77.8% said they surf the Internet to learn more about their medical condition.

**Table 1:** Demographic characteristics of the participants (N = 424).

Variables		n (%)
Age	Less than 30	167 (39.4)
	30–40	122 (28.8)
	More than 40	135 (31.8)
Sex	Male	185 (43.6)
	Female	239 (53.4)
Marital status	Married	207 (48.8)
	Single	217 (51.2)
Nationality	Saudi	306 (72.2)
	Non-Saudi	118 (27.8)
Place of residence	Urban	357 (84.2)
	Rural	67 (15.8)
Region in Saudi Arabia	Riyadh (Central Region)	120 (28.3)
	Makkah province (Western Region)	94 (22.2)
	Eastern Province (Eastern Region)	80 (18.9)
	Aljouf province (Northern Region)	70 (16.5)
	Asser province (Southern Region)	60 (14.2)
Are you a frequent user of social media?	Yes	333(78.5)
	No	91 (21.5)
Do you surf the internet to learn more about your medical condition?	Yes	330 (77.8)
	No	94 (22.2)

Table 2 presents the means and standard deviations (SD) of the study variables. The mean BRIEF, CSS, and SHAI scores were 12.30 ± 2.65, 36.01 ± 8.13, and 22.10 ± 9.52, respectively.

**Table 2:** Mean and SD of BRIEF, CSS, and SHAI scores.

Variable		Mean±SD
BRIEF		12.30 ± 2.65
CSS		36.01 ± 8.13
	Excessiveness	9.85 ± 2.24
	Distress	8.76 ± 2.70
	Reassurance	9.01 ± 2.51
	Compulsion	8.37 ± 2.76
SHAI		22.10 ± 9.52
	Health Anxiety (items 1–14, range 0–42)	17.57 ± 7.52
	Negative Consequences’ of becoming ill (items 15–18, range 0 to 12)	4.53 ± 2.70

Table 3 presents the factors affecting the BRIEF, CSS, and SHAI scores. The results show that Age was associated with BRIEF (p < 0.001), CSS (p < 0.001), and SHAI (p < 0.0010). Sex, especially female sex, was associated with SHAI (p < 0.026), and rural place of residence was associated with CSS (p < 0.001). The Makkah, eastern, and Aljouf regions were associated with SHAI (p < 0.001), CSS (p < 0.001), and BRIEF (p < 0.001), respectively. The status of not being a frequent user of social media was associated with CSS (p < 0.001), whereas not being an Internet user was associated with CSS (p < 0.001).

**Table 3:** Associations between demographic and behavioral factors and BRIEF, CSS, and SHAI scores.

			BRIEF			CSS			SHAI	
		β	95% CI for β	Sig.	β	95% CI for β	Sig.	β	95% CI for β	Sig.
Age		−0.094	−.131−.056	<0.001	0.128	0.065–0.191	<0.001	–.246	–.381–.111	<0.001
Sex	Male	N/A			Reference			Reference		
	Female				−0.304	–1.633–1.025	0.653	2.153	.260–4.045	0.026
Marital status	Married	Reference			N/A			Reference		
	Single	–0.941	NA	0.014				−0.256	–2.739–2.227	0.839
Nationality	Saudi	Reference			N/A			Reference		
	Non-Saudi	0.392	–.422–1.205	0.344				.920	–2.144–3.983	0.555
Place of residence	Urban	Reference			Reference			Reference		
	Rural	–0.264	–1.061–.532	0.514	–8.343	–10.466–6.220	<0.001	–5.162	–7.803–2.521	<0.001
Region	Riyadh (Central Region)	Reference			Reference			Reference		
	Makkah province (Western Region)	.164	–.715–1.044	0.714	–2.796	–4.738–.853	.005	–7.735	–10.171–5.299	<0.001
	Eastern Province (Eastern Region)	–.724	–1.652–0.205	0.126	8.461	6.249–10.673	<0.001	.557	–2.057–3.170	.676
	Aljouf province (Northern Region)	1.719	0.747–2.691	0.001	−.035	–1.788–1.718	.969	–6.171	–8.407–3.935	<0.001
	Asser province (Southern Region)	−0.469	–1.297–0.360	.267	.824	–1.390–3.038	.465	–2.416	–5.337–.505	0.105
social media	Yes	N/A			Reference			Reference		
	No				–4.014	–6.046–1.983	<0.001	–3.692	–6.238–1.146	0.005
internet	Yes	N/A			Reference			Reference		
	No				–2.306	–4.066–.545	0.010	–1.192	–3.201–.817	0.244

BRIEF R=0.395, R square=0.156, adjusted R square=0.141, F=10.498, p-value<0.001.

CSS R=0.616, R square=0.379, adjusted R square=0.367, F=30.714, p-value<0.001.

SHAI R=0.545, R square=0.297, adjusted R square=0.280, F=17.298, p-value<0.001.

SEM revealed that cyberchondria had an effect on HL and health anxiety (p < 0.001). Additionally, HL had an effect on health anxiety (p < 0.001).

**Fig 1:**
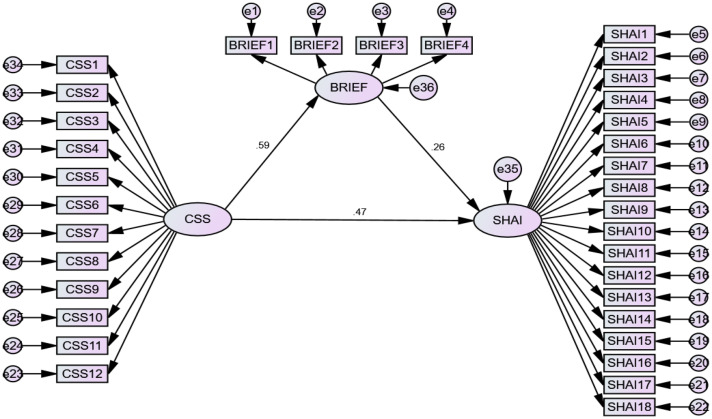
Emerging model of the influence mediating role of health literacy in the link between cyberchondria and health anxiety. The model depicted here is an original result of the study, illustrating the significant pathways identified through mediation analysis. Abbreviations: CSS, Cyberchondria Severity Scale; BRIEF, health literacy scale; SHAI, Short Health Anxiety Inventory.

**Regression Weights: (Group number 1 – Default model)** Fig 1

**Table:** 

			Estimate	S.E.	C.R.	*P*
BRIEF	<---	CSS	0.378	0.071	5.351	***
SHAI	<---	BRIEF	0.390	0.109	3.577	***
SHAI	<---	CSS	0.456	0.067	6.802	***

## Discussion

This study aimed to investigate the prevalence of cyberchondria among primary healthcare patients in Saudi Arabia and to explore the relationship between cyberchondria, health anxiety, and health literacy. In this study, the average scores for BRIEF, CSS, and SHAI among the sample population indicated low health literacy, moderate levels of cyberchondria, and moderate levels of health anxiety, respectively. These findings imply that participants in this study could be more susceptible to misleading information, and illness-related stress [33] explained that reduced health literacy can worsen the condition by worsening health anxiety, which increases cyberchondria risk. This concurs with the current findings, which infer that people with less capacity to take care of their health may be predisposed to these issues. In addition, Starčević et al. [34] observed a correlation between cyberchondriasis and rising levels of health anxiety. Thus, it underpins the concept that excessive online health information seeking can increase anxiety levels. Pelle et al. (2020) [35] indicated that technology-based interventions can result in more positive patient outcomes, especially among those suffering from osteoarthritis, as an example of such conditions. This shows that through technological solutions, there are some ways to deal with a lack of knowledge regarding good health, cyberchondria, and hypochondria. To effectively address low health literacy rates among patients, reduce the incidence or implications of cyberchondria, and deal with case studies tailored to mitigate heightened feelings of terror about one’s well-being, it is very important for healthcare providers to come on board to champion such causes. Inclusion involves developing programs for the critical appraisal of online medical resources alongside counseling and supporting individuals who have medical anxieties to alleviate these problems. To this end, engagement within the healthcare sector may also involve encouraging accountable usage technology within healthcare, while developing e-tools designed to assist self-management among patients.

A noticeable trend in the data indicated that individuals below 30 years of age had considerably lower BHLS scores than their older counterparts did. This means that the new generation is more likely to search for health information as they know how to use the Internet. Wallston et al. [34] supported this finding, which was obtained from a study of clinical nurses and showed that people aged below 39 are more likely to seek health information online than those aged 45 years or older. Government organizations should establish an all-inclusive site to address specific diseases, as proposed earlier. Young people would have immediate access to reliable and up-to-date medical data with respect to this platform as a central hub. Such websites may cover various matters concerning different diseases, including their symptoms, causes, treatment options, and prevention methods.

Compared to younger adults, older people aged 40 years and above showed much higher CSS scores. Some possible explanations include vulnerability to chronic diseases that are common with aging, cognitive changes that occur with aging, and social isolation. In this regard, a recommended monthly checkup for the elderly is advisable. These results support those of Kirkman et al. [36] and Maresova et al. [37], who noted that the elderly were more inclined to search for health services online, especially those showing signs of cognitive impairment or high levels of anxiety. To address this particular problem among elderly people, it is suggested that medical institutions should have regular monthly check-ups for this age group. Such a strategy would greatly enhance their general well-being and improve their health status.

The pervasiveness of technology has been thought to greatly affect the lifestyles of young people, who are often obsessed with their smartphones and other social media platforms. This constant connectivity results in less face-to-face interaction, which limits the expression of emotions and possibly increases anxiety. Alongside the vast amount of health-related information available online, it enhances anxiety as well as cyberchondria. This is confirmed by the greater scores commonly seen among younger adults on the SHAI compared with older individuals. These findings indicate that there is a lot of online health information that may increase anxiety because people ever encounter perceived threats to their safety. A study conducted by Abramowitz et al. [38] supports these claims by demonstrating how younger adults get higher SHAI scores due to media and social platforms, which expose them to more health-related information. Therefore, government institutions need to have recurring monthly activities to engage the youth in productive initiatives. Issuing regular monthly initiatives aimed at keeping the younger generation busy would help solve this problem. For instance, such interventions could promote socialization or provide an opportunity through which they can direct their energy positively, thus limiting the negative effects brought about by the extreme consumption of online health materials.

Regarding sex and SHAI, there was a strong correlation between females and higher inventory scores. This finding implies that females have significantly greater scores on the SHAI, indicating that gender might be one of the factors contributing to the development of health anxiety. The possible causes of this difference include complex relations within society, biological determinants, and attitudes towards asking for help during illness. It is recommended that research be carried out to isolate these effects and determine specific factors leading to this divergence in health fears between males and females. The above results are consistent with the argument of Macswain et al. [39], suggesting that women tend to express their thoughts openly when compared to men, which demonstrates their natural propensity for effective communication and emotional expression. Thus, medical practitioners should acknowledge such distinctions in gender as they adjust their strategies. Additionally, supporting openness and emotional expressiveness among women might contribute to reducing the rates of health anxiety among them.

Geographical location also significantly influenced CSS scores of people in rural areas. These constraints prevent individuals from receiving timely and high-quality care, leading to cyberchondria development. However, existing literature does not specifically state whether people in rural areas have experienced cyberchondria. This research examines what contributes to “Dr.Google” consultations and health anxiety among primary care patients. It further examined the role of HL as a potential mediator between these variables. The results show that young adults differ from older adults in terms of access to health information and levels of cyberchondria. In addition, it shows how technology affects mental well-being in younger age groups, as well as the influence of gender on health anxiety. Finally, this study highlights some of the problems faced by those living in the countryside while accessing healthcare services as well as authentic sources of information. Earlier studies have supported this idea by demonstrating a relationship between health anxiety and cyberchondria, suggesting that excessive online health information seeking increases levels of health anxiety [40]. Moreover, internet use for health-related matters is prevalent among individuals with symptoms related to HA, thereby worsening cyberchondria [41]. Studies have shown that during the COVID-19 pandemic, there was increased distress associated with compulsive behaviors related to medical inquiries [42].

The observed connection between rural residence and high CSS scores points to an intricate relationship between geographical, socioeconomic, and access to healthcare factors. People in such areas often have a hard time getting timely medical care that is sufficient because of the long distances they have to travel as well as a lack of specialists and financial limitations. Anxiety levels increase due to these challenges, leading to increased use of Internet information related to personal diagnosis and treatment decision-making. A broader approach towards tackling this issue should include, but not be limited to, better healthcare infrastructure, enhanced telemedicine services, and digital literacy among rural people. This is because it has been found that anxiety levels in regions with inadequate healthcare and Internet access are higher, leading to the development of cyberchondriac [43,44]. To mitigate this problem, government health institutions should undertake initiatives such as extending healthcare services into rural areas, boosting internet connectivity, enhancing HL and education, promoting face-to-face interaction and social engagement, and creating awareness among healthcare providers on cyberchondria. Remarkably, people living in the Makkah region of Saudi Arabia made a major contribution to SHAI scores. It is true that Makkah is a holy city for Muslims, and it attracts many pilgrims along with tourists who have the opportunity to use the Internet and receive medical services. However, owing to its large population size, there can be significant delays in timely care due to the increased demand for health services. In these cases, long waits may lead to higher scores on the SHAI as people become increasingly worried about their loved ones. On the other hand, the eastern province of Saudi Arabia has a high number of industries, including petrochemical plants, besides being one of the richest oil-producing provinces that account for the special features of CSS values here. The presence of these industries may expose individuals to pollutants and other environmental hazards, potentially increasing the risk of health issues. Moreover, region’s hot and humid climate could also serve as another factor increasing stress levels among residents hence affecting their CSS scores Accordingly, environmental factors, such as extreme heat and humidity, have been associated with increased stress levels and adverse mental health outcomes [45] These conditions may contribute to heightened anxiety and subsequent cyberchondria behaviors as individuals seek online health information to cope with perceived threats to their well-being.

To prepare for the healthcare needs of Makkah’s growing population, existing facilities should be expanded along with mobile health centers dispatched during peak seasons. These measures could help reduce waiting times and provide additional medical services for pilgrims and tourists. In the Eastern region, community-based health education programs should be implemented to raise awareness about the health risks associated with industrial pollution. Additionally, encouraging regular health check-ups and screenings could aid in the early detection of potential health problems and provide timely interventions. In the Aljouf region, the expansion of healthcare infrastructure and telemedicine access is crucial to improving access to quality healthcare services. A recent study reported a correlation between CSS scores and individuals who were infrequent social media users [46]. This suggests that those who do not rely heavily on social media may seek health information from their relatives or friends, potentially leading to misconceptions. Although this requires more research for definitive results, a study by Chen and Wang [46] indicated that social media platforms encourage patients to make greater use of the healthcare system. The relationship among CSS scores, social media use, and health information-seeking activities requires further study. Furthermore, exploring the factors influencing infrequent social media users’ inclination to seek health information from their social circles rather than relying on social media could help inform strategies to encourage this population’s use of reliable online health resources.

An SEM finding of interest is the impact of cyberchondria on both HL and health anxiety. This implies that some health information found on the internet or social media platforms may not be applicable to a person’s specific health problems or conditions. Consequently, HL for the Internet and social media followers is directly influenced by it, as they depend wholly on what they find on these websites. Failure to understand or misinterpret this information can lead to high levels of health anxiety, similar to a serious medical condition. It has recently emerged as a subject of research interest concerning the relationship between cyberchondria, health literacy (HL), and health anxiety. Some studies have shown that excessive searching for health information online, which describes cyberchondria, may increase the risk of developing high levels of health anxiety [47]. This idea becomes more significant among people who spend most of their time on the web because such individuals tend to misrepresent online medical details, thereby fueling their fears about disease [47]. The mediating effect indicates even further complexity in the association between cyberchondria and HL, which suggests intricate interplay among them [33,36]. To tackle the problems associated with cyberchondria, suggestions include having digital literacy programs while partnering with social media channels where accurate healthcare-promoting data can be availed [33]. By cutting down on excessive online health information quests and teaching people to be critical of what they come across, it is possible to boost HL and reduce the negative effects of cyberchondria on health anxiety [33]. Further, other interventions could combine mindful techniques with healthy habits so that individuals would have a more insightful approach to health-related internet data that would help in managing fears and trauma [33]. Additionally, previous studies have emphasized the importance of understanding the diverse ways social media affects mental health especially anxiety [48]. Anxiety has been reported as one of several mental disorders associated with high levels of depression and increased instances of fearfulness [49]. Moreover, individuals with high levels of support from their peers experience a good mental state through social media [50]. Therefore, holistic anxiety management strategies should acknowledge the complex relationships between social media and mental health that can promote psychological well-being [51]. Therefore, there is a need for intervention programs to improve digital literacy and develop critical appraisal skills for online healthcare information. However, this will help them pass through this lot of information in smart gadgets or other appliances linked to the Internet, thereby making it possible to lower pressure points and simultaneously create resilience concerning health ambiguities.

### Implications for practice

The research suggests that BRIEF, health literacy, and anxiety are salient factors in cyberchondria and health anxiety. Hence, holistic intervention is required. Coping techniques, such as those that improve stress management skills, can decrease the tendency toward cyberchondria, whereas high health literacy assists people in critically analyzing online health content. Cognitive-behavioral interventions, along with other relaxing exercises such as mindfulness, would also lower levels of anxiety. Moreover, if demography is well thought-out when planning focused interventions, it will further enhance the outcomes by considering characteristics such as age, gender, and place of residence. Therefore, there is a need for extensive programs aimed at preventing or reducing cyberchondria and health anxiety fashioned by healthcare providers together with educators and policymakers alike.

In addressing cyberchondria, health care providers’ top priority is to promote health literacy. This can be achieved by having open and honest conversations with patients that allow them to see cyberchondria ahead of time, thus recognizing its potential dangers and directing it as appropriate. It involves acknowledging fear, validating feelings, and providing accurate information. Educating people about the limitations of online health information and the importance of consulting experts are also part of this endeavor. Similarly, medical practitioners can use simple language when talking and encourage patients to ask queries to boost their health literacy. The availability of resources and educational materials enables people to make informed decisions concerning their state of wellness, thereby promoting their overall well-being. Hence, establishing mutual trust and respect between patients and caregivers is very important for mitigating the risks associated with cyberchondria, it also assists in building a culture of good health among the general population.

Future investigations need to explore interventions that can improve health literacy and lessen health anxiety in people who rely on the internet for medical information. It could involve tailored educational programs designed, credible online platforms with pertinent health information, and an assessment of the efficacy of different communication methods. Furthermore, there is also a need for studies that determine how cyberchondria and health anxiety affect the long-term quality of life of individuals, thereby impacting their healthcare utilization in the process. These gaps in knowledge will help researchers develop better ways of preventing and intervening.

### Limitations

It should be noted that this study was cross-sectional in nature, which means it did not establish cause-and-effect relationships. Future longitudinal studies are required to identify linkages between the variables under investigation. Furthermore, the research was carried out in Saudi Arabia; hence, the results may not apply to other populations. Notwithstanding these limitations, this study offers important information about cyberchondria and its correlation with health anxiety. Consequently, they can help draft measures to prevent or treat them.

## Conclusion

Health literacy, cyberchondria, and health anxiety are interlinked with demographic and behavioral influences. These findings underscore the significance of health literacy interventions in reducing health anxiety and mitigating cyberchondria. These findings indicate that cyberchondria is a complex phenomenon with numerous attributes. This study also showed that one can develop health anxiety from cyberchondria.

## Supporting information

S1 File Dataset. (S1 File.Dataset.XLSX)
